# Combination of Red Clover and Hops Extract Improved Menopause Symptoms in an Ovariectomized Rat Model

**DOI:** 10.1155/2020/7941391

**Published:** 2020-05-23

**Authors:** Mi Ran Kim, Hyun Jin Kim, Su Hyun Yu, Bo Su Lee, Se Yeong Jeon, Jeong Jun Lee, Young Chul Lee

**Affiliations:** Natural Product Team, Naturech Co, Ltd., Chungcheongnam-do 31257, Republic of Korea

## Abstract

Red clover and hops are already known for their alternative menopausal therapies; however, their combination has not yet been studied. This study aimed to evaluate the efficacy of the combination of red clover and hops extract (RHEC) for treating menopausal symptoms for the first time. A high-performance liquid chromatography (HPLC) method for RHEC was developed and validated for the analysis of biochanin A in red clover extract and xanthohumol in hops extract. An in vivo study was conducted using an ovariectomized rat model treated with RHEC (125, 250, and 500 mg/kg, p.o.) for a 12-week test period. Changes in body weight, tail skin temperature (TST), serum lipid profile, bone metabolism, antioxidants, and markers of vasorelaxation and uterus endometrium were evaluated. RHEC significantly inhibited body weight gain and decreased fat weight. Changes in TST associated with flashes were significantly inhibited in the RHEC groups. Other markers related to menopausal symptoms, such as blood lipid profile (total cholesterol and low-density-lipoprotein cholesterol), bone metabolism (serum alkaline phosphatase, osteocalcin, and c-terminal telopeptide type 1), antioxidants (superoxide dismutase and malondialdehyde), and vasorelaxants (endothelin-1 and nitric oxide), were significantly improved after the administration of RHEC. We also confirmed the safety of RHEC through histopathological observation of the endometrium. Our findings demonstrate that RHEC appears to have high potential for comprehensively improving various symptoms of menopause.

## 1. Introduction

Menopause is a natural process that develops in women typically in their late 40s and involves a decrease in the estrogen levels, particularly due to ovarian decline [1, 2]. This decrease in estrogen levels is accompanied by various symptoms, such as hot flashes, night sweats, and depression [3]. Estrogen deficiency can lead to an increased risk of metabolic diseases, such as obesity, heart disease, diabetes, and hypertension [4–6]. Furthermore, it affects the atherogenic lipid profile, such as the levels of high-density-lipoprotein (HDL) and low-density-lipoprotein (LDL) cholesterol [7].

Hormone therapy is the main treatment option for menopausal symptoms; however, long-term treatment via this approach can cause cardiovascular events and breast cancer [8]. Concerns about the safety of hormonal products have increased the popularity of many complementary therapies such as red clover extract. Various menopausal symptoms reduce the quality of life of menopausal women, so it is important to manage these symptoms healthily and properly through dietary supplements that lack side effects.

Red clover (*Trifolium pratense* L.) has estrogenic effects due to the presence of isoflavones, which are plant-based chemicals having estrogen-like effects on the body [9]. Thus, red clover botanical dietary supplements have been used for treating menopausal symptoms and for maintaining or improving cardiovascular health. The main isoflavones of red clover are biochanin A, formononetin, ononin, and sisotrin. Upon ingesting red clover, biochanin A and formononetin are demethylated and metabolized to genistein and daidzein in the body [10].

Hops (*Humulus lupulus* L.) are the main ingredient used to brew beer and has been traditionally used in Europe to facilitate sleep and reduce mental stress. Hops contain phytochemicals with a wide range of biological activities, including chemopreventive, cardioprotective, and anti-inflammatory effects [11]. Hops dietary supplements have been studied as botanical alternatives to hormone therapy for relief from hot flashes and related symptoms in menopausal women [12]. The main components of hops are xanthohumol (chalcone), isoxanthohumol (flavone), and 8-prenylnaringenin (prenylated flavonoid) [13, 14].

Based on the abovementioned information, we expect that the combination of red clover extract and hops extract (RHEC) can improve the various symptoms of the menopause. In this study, we investigate the effects of RHEC on tail skin temperature (TST), serum lipid profile, bone metabolism, antioxidant, and markers of vasorelaxation using an ovariectomized model (OVX) that displays a decline in estrogen levels similar to that in menopausal women.

## 2. Materials and Methods

### 2.1. RHEC Preparation

The red clover (leaf) and hops (flower and flower buds) used in the experiment were purchased from a herbal market (Danyang, China). The method of extraction was as follows: red clover (200 g) was extracted three times with 70% ethanol (85°C and 3 h reflux). The extract was filtered through a filter paper and concentrated under reduced pressure; the concentrate was precipitated at 4°C for 24 h and then the precipitate was dried. Hops (200 g) were extracted twice with 70% ethanol (85°C and 4 h reflux). The extract was filtered through a filter paper, concentrated under reduced pressure, and then dried. Extracts were mixed at a weight ratio of 3 : 1 and used to assess in vivo efficacy.

### 2.2. High-Performance Liquid Chromatography (HPLC) Analysis of RHEC

An Agilent HPLC 1200 series system equipped with a quaternary pump, autosampler, and photodiode array detector with a Phenomenex® Luna C18 column (250 × 4.6 mm, 5 *μ*m) was used for analysis (Phenomenex Inc., Torrance, CA, USA). Elution with solvent A (acetonitrile) and solvent B (0.25% formic acid in water) in a gradient elution at a flow rate of 1 mL/min was carried out as follows: 0–3 min, 75% B; 3–33 min, 75%–30% B; 33–40 min, 30%–10% B; 40–45 min, 10% B; 45–45.5 min, 10%–75% B; and 45.5–50 min, 75% B. The detection wavelength was set at 320 nm. The column temperature was kept at 30°C and the injection volume was 10 *µ*L.

### 2.3. Animals and Treatment

The study was approved by the Institutional Animal Care and Use Committee of Naturetech, Inc. (approval no. NT 11803), based on the National Animal Welfare Law of the Republic of Korea. Thirty-five virgin female Sprague-Dawley rats (7 weeks old) were obtained from OrientBio Co., Ltd. (Seongnam-si, South Korea). They were acclimated to the laboratory conditions for a week and housed in a specific pathogen-free room at a standard temperature of 24.0°C ± 1.0°C with a humidity of 50% ± 10% under a 12 h light/dark cycle. Water and feed (ENVIGO 2018s, Envigo, Madison, WI, USA) were given ad libitum. One week after arrival, the rats were anesthetized using pentobarbital sodium (40 mg/kg) intraperitoneally and subjected to a bilateral ovariectomy (OVX) or sham operation via double dorsolateral incision. The experimental groups were divided into the following five groups (7 rats per group): Sham and OVX and RHEC 125, 250, and 500 mg/kg groups. At 4–5 days after surgery, ovariectomized animals received red clover and hops extract combination (RHEC 125, 250, and 500 mg/kg, p.o.) and vehicle (distilled water) for 12 weeks. The body weights of all animals were examined at the beginning of the experiment and at weekly intervals throughout the 12-week experimental period. BW gain was calculated as the difference between initial and final body weights. Food consumption was recorded during the 12-week period of RHED administration on a weekly basis and the mean daily food consumption was calculated using the total. At the end of the experiment, rats were anesthetized with 2%–3% isoflurane and blood samples were collected from the ventral aorta. The uterus, liver, kidney, and fat pads (visceral and retroperitoneal) were dissected out and weighed. Uteri were fixed in 10% buffered formaldehyde and blocked in paraffin. The paraffin block was cut into 3–5 *µ*m sections and stained with hematoxylin and eosin for light microscopic analysis.

### 2.4. Measurement of TST

On the day of measuring TST, vehicle or RHEC was administered orally 30 min before the measurements.

#### 2.4.1. Under Natural Conditions

The rats were removed from the cage and placed on an experimental table. Then, only the tail was lightly held, and the temperature of the dorsal part of the tail about 2 cm away from the base was measured using an infrared thermometer (Model 153IRB, Bioseb, Chaville, France). After a total of three measurements, the average value was obtained.

#### 2.4.2. Under Stressed Conditions

TST under stressed conditions was measured using a slightly modified method as previously described [15]. Rats were restrained in a holder in a conscious condition and TST was measured for 1 h on the dorsal side of the tail about 2 cm from the fur line using an infrared thermometer. Before testing, all animals were allowed to acclimate to the laboratory for 15 min. The ambient temperature was 25°C. TST data were measured at 10 min intervals throughout the experimental period.

### 2.5. Serum Analysis

Blood samples were centrifuged at 3,000 rpm and 4°C for 15 min; serum samples were collected and stored at −70°C until analysis. Alanine aminotransferase (ALT), aspartate aminotransferase (AST), alkaline phosphatase (ALP), total cholesterol (T-Chol), triglyceride (TG), low-density-lipoprotein cholesterol (LDL-C), and high-density-lipoprotein cholesterol (HDL-C) were measured using an automatic blood chemistry analyzer (BS-220, Mindray, Xuzhou City, China). Measurements of osteocalcin, C-telopeptide of type I collagen (CTX-1), superoxide dismutase (SOD), malonyl dehydrogenase (MDA), endothelin-1, and nitric oxide in serum were performed using the Rat Osteocalcin ELISA kit (Cusabio Biotech, Houston, TX, USA), RatLaps (CTX-1) EIA Assay (Immunodiagnostic System, Gaithersburg, MD, USA), OxiSelect™ Superoxide Dismutase Activity (Cell Biolabs, Inc., San Diego, CA, USA), OxiSelect™ TBARS (Thiobarbituric Acid Reactive Substances) Assay Kit (MDA Quantitation, Cell Biolabs Inc., San Diego, CA, USA), Quantikine ELISA Endothelin-1 kit (R&D Systems, Minneapolis, USA), and colorimetric NO assay kit (R&D Systems, Minneapolis, MN, USA), following the manufacturer's instructions. All samples were analyzed in duplicate in the assay.

### 2.6. Statistical Analysis

All statistical analyses were performed by one-way ANOVA using SAS software (SAS Institute Inc., Cary, NC, USA). The repeated measure was used for statistical analysis of the levels of body weight and TST over time. Multiple comparisons were performed with Duncan's multiple-range tests and Tukey–Kramer adjustment. Results are presented as mean ± SD. Values of *P* < 0.05 were considered to represent statistical significance.

## 3. Results

### 3.1. HPLC Analysis of RHEC

**Figure 1:**
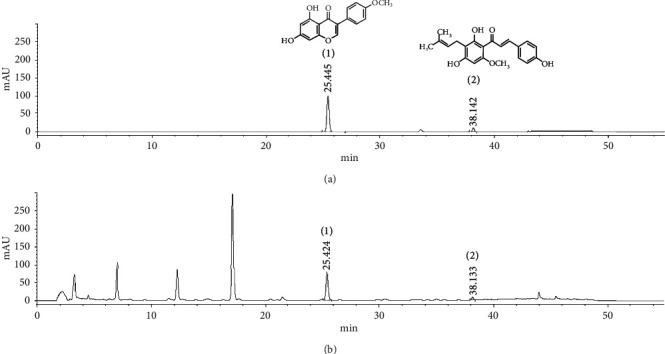
Representative HPLC chromatogram at 320 nm: standard solution (a) of biochanin A (1) and xanthohumol (2) and RHEC (b).

Based on the absorption and retention time of standard materials, biochanin A and xanthohumol (Figure 1(a)) were identified as major components of RHEC, and the results confirmed that RHEC contained 10.3 ± 0.9 mg/g biochanin A (25.4 min) and 0.3 ± 0.04 mg/g xanthohumol (38.1 min) (Figure 1(b)).

### 3.2. Body and Fat Weights

**Figure 2:**
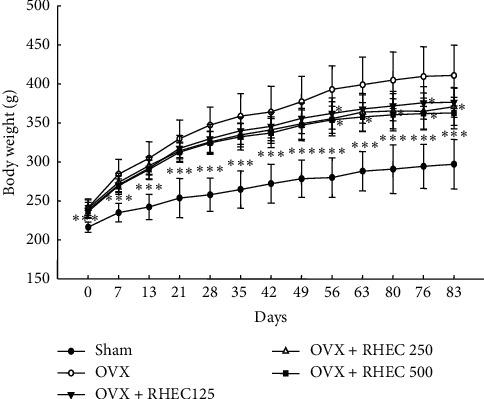
Changes in body weight during 12 weeks of RHEG administration. Values are presented as mean ± SD (*n* = 7). ^*∗*^*p* < 0.05,^*∗∗*^*p* < 0.01, and^*∗∗∗*^*p* < 0.001 versus OVX.

The changes of mean body weight throughout the experimental period are reported in Figure 2. The OVX group showed significantly increased weight change compared with the sham group. Oral administration of RHEC to ovariectomized rats for 12 weeks was observed to inhibit body weight gain during the entire experimental period. After 8 weeks of administration of RHEC at 500 mg/kg, there was significant inhibition of the increase in body weight. In addition, body weight gain was significantly reduced at the end of the experiment in all treatment groups. Furthermore, after RHCE administration, the visceral and retroperitoneal fat weights were significantly decreased when compared with that in the OVX group. Feed intake in the OVX group was significantly higher than in the sham group but significantly decreased in the 120 mg/kg RHEC group. The 250 and 500 mg/kg RHEC groups also slightly decreased, but no significance was observed (Table 1).

### 3.3. Tail Skin Temperature (TST)

**Figure 3:**
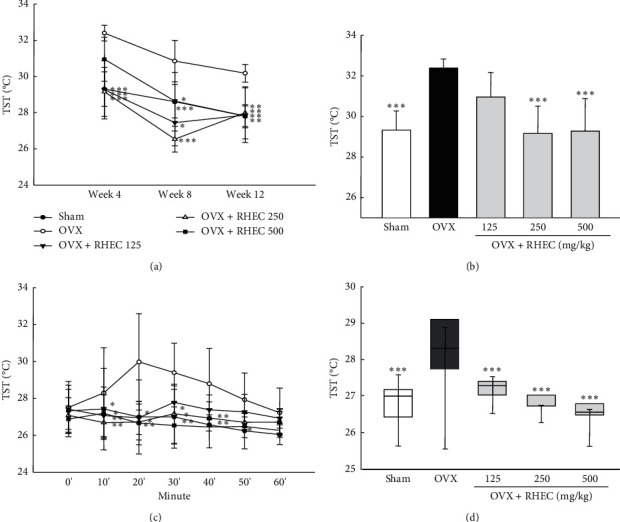
Results of tail skin temperature (TST) measured under natural conditions from weeks 4 to 12 (a) and TST in Week 4 (b). TST under the stress conditions for 1 h (c) and temperature range during 1 h (d). Values are presented as mean ± SD (*n* = 7). ^*∗*^*p* < 0.05,^*∗∗*^*p* < 0.01, and^*∗∗∗*^*p* < 0.001 versus OVX.

Measurements of TST under natural conditions in weeks 4, 8, and 12 are shown in Figure 3(a), and detailed changes in TST on Week 4 are shown in Figure 3(b). The OVX group showed significant increases throughout the experiment compared with the sham group. The increase in TST by ovariectomy was markedly inhibited by the administration of RTEC. In addition, under the stress conditions, the OVX group showed significantly increased TST at 20–60 min after measurement (Figure 3(c)). However, TST in RHEC rats (125, 250, or 500 mg/kg) was significantly (*P* < 0.05) lower than that of the OVX group. In addition, regarding the change of TST during 60 min, the temperature of the OVX group was significantly more elevated than that of the sham group and the RHEC groups showed a decreased range of TST in a dose-dependent manner (Figure 3(d)).

### 3.4. Lipid Profile

The OVX group showed significant elevations of total cholesterol, HDL-C, and LDL-C. After 12 weeks of administration of RHEC, total cholesterol significantly decreased in the RHEC 500 mg/kg group. Triglyceride also decreased in all treatment groups, albeit not significantly (Table 2).

### 3.5. Bone Metabolism-Related Biomarkers

Serum ALP, osteocalcin, and CTX-1 were significantly increased in the OVX group. However, in the RHEC-treated groups, these markers showed patterns of decreases in a dose-dependent manner; CTX-1 in particular was significantly decreased in the three dose groups (Table 2).

### 3.6. Antioxidant Capacity

The OVX group showed a decrease in SOD level in serum and an increase in MDA level when compared with the sham group (Table 2). However, the administration of RHEC at 500 mg/kg for 12 weeks significantly restored these levels.

### 3.7. Change in Vasorelaxation Markers

**Figure 4:**
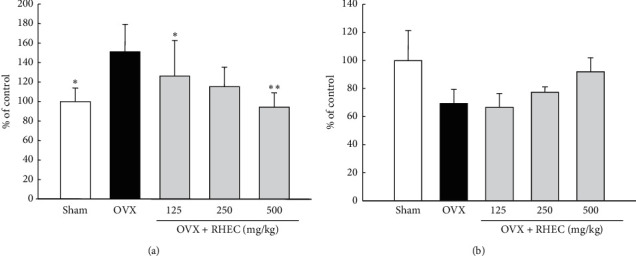
Results of endothelin-1 (a) and nitric oxide (b) in serum. Values are presented as mean ± SD (*n* = 7). ^*∗*^*p* < 0.05,^*∗∗*^*p* < 0.01, and^*∗∗∗*^*p* < 0.001 versus OVX.

In the OVX group, plasma endothelin-1 was significantly increased when compared with the level in the sham group (Figure 4(a)), but nitric oxide was decreased (Figure 4(b)). However, these changes were significantly normalized by the administration of RHEC and showed a dose-dependent result (Figure 4).

### 3.8. Safety Evaluation of RHEC

**Figure 5:**
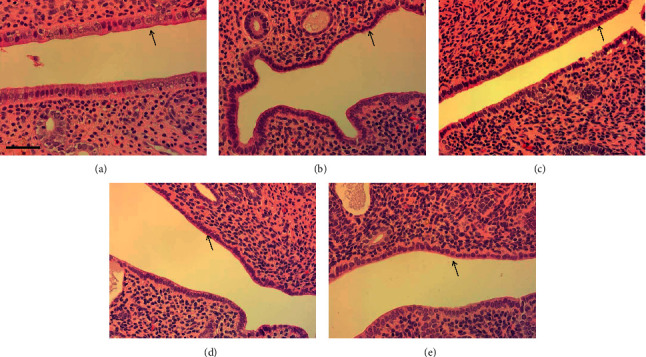
H&E staining of the uterus. Sham (a), OVX (b), RHEC 125 mg/kg (c), RHEC 250 mg/kg (d), and RHEC 500 mg/kg (e). Black arrows indicate the epithelial layer of the endometrium. The bar indicates 50 *µ*m (magnification × 200).

After 12 weeks of administration of RHEC, organ weights of the liver and kidney (Table 1) and serum levels of ALT and AST (Table 2) did not show any differences. OVX rats showed the atrophy of endometrial epithelial cells, and RHEC groups showed the same microscopic findings as that of the OVX group (Table 1 and Figure 5); there were also no differences in uterus weights among the OVX and RHEC groups (Table 1).

## 4. Discussion

The estrogenic component found in plants is called phytoestrogen, which is present at an especially high level in *Fabaceae*. It exhibits activity similar to that of estrogen and is mostly a flavonoid and phenolic acid substance [16]. One of the most studied phytoestrogens is isoflavone. The isoflavone component of red clover binds to the estrogen receptor and is reported to significantly alleviate estradiol reduction associated with menopause when ingested with red clover [17, 18]. Xanthohumol, isoxanthohumol, and 8-prenylnaringenin (8-PN) in hops are also known as phytoestrogens [19]. Red clover and hops extracts have been reported to improve the fragmentary symptoms of menopause, such as osteoporosis and hot flashes; however, their effectiveness is still being debated [20–22]. Building on this previous work, the present study was conducted to study the overall improvement of women's menopausal symptoms, such as accumulation of body fat, hot flashes, abnormal blood lipids, osteoporosis, and risk of CVD, by the administration of RHEC in OVX rats.

The synergistic effect of RHEC on the estrogen receptor was confirmed in our previous study (*data in progress-under submission*), and in this study, we confirmed its improvement of various symptoms associated with estrogen deficiency using an ovariectomized rat model.

Similar to a report describing that the reduction in estrogen secretion by ovarian resection causes weight gain [23], the body and fat weights of the rats in the OVX group were higher than that in the sham group. RHEC administration inhibited weight gain and abdominal fat accumulation, and this effect is similar to that seen after estrogen treatment in OVX rats [24]. This weight loss was not observed in the red clover alone [25]; weight loss was observed in high contents of xanthohumol in hops [26, 27]. Biochanin A and xanthohumol contents in RHEC were approximately 1.09% and 0.033%, respectively. Although RHEC showed similar or lower contents than the active compounds mentioned in previous studies [25, 26], it showed weight gain inhibition and abdominal fat accumulation effects. Because we have already confirmed the effects of estrogen receptor activity on RHEC through in vitro tests (*data in progress-under submission)*, this effect is expected to be via estrogen receptor activity.

One of the main symptoms of menopausal women, hot flashes, is a temporary symptom involving severe fever of the neck and face, which causes sweating and chills and greatly reduces the quality of life. The precise mechanism behind hot flashes is unclear, but they are speculated to be caused by dysfunction of the autonomic nervous system caused by rapid heat loss due to increased peripheral blood flow and skin conductivity [28]. In addition, estrogen is considered to play an important role in hot flashes because they appear during menopause, when blood levels of estrogen are low [29]. TST was measured in this experiment. In the RHEC group, an increase in TST was significantly suppressed under normal as well as under stress conditions. It was confirmed that TST decreased when 8-PN, one of the main components of hops, was administered to OVX rats [30]; moreover, it was found that changes in TST and rectal temperature decreased upon consumption of Lifenol® [27], a standardized hops CO_2_ extract. While the clinical test results of red clover for the iris have been abundantly reported [31], there are few reports about TST results in animals. In this study, we found that the TST range of fluctuation by physical stress decreased in the RHEC group, suggesting that the interaction of RE and HE may affect central thermoneutral and peripheral vasodilation.

Estrogen deficiency is involved in the development of cardiovascular disease (CVD) in women, and it plays a role in endothelial function and vascular tone, among others [32]. Newson [33] recommends early and active prevention and response for managing cardiovascular risk factors. Therefore, the prevention of cardiovascular risk factors is considered one of the main indicators when evaluating the efficacy of RHEC. In this study, the levels of TC, TG, HDL-C, and LDL-C related to cardiovascular disease were measured and the levels of ET-1 and NO were analyzed in the blood to evaluate endothelial function. The results showed significant reductions in T-Chol in the RHEC 500 mg/kg dose group, along with tendencies for reduction in the levels of TG and LDL-C. These results are similar to those observed in dyslipidemia due to estrogen reduction being alleviated by estrogen replacement therapy [34]. Furthermore, RE and HE have been observed to have efficacy related to lipid metabolism. A clinical study reported that TC and LDL-C levels decreased during the 3-month intake of RE [35] and that HDL-C levels increased after 1 year of administration [36]. Many studies have also reported about a link between menopausal conditions and elevated blood lipid levels [37].

Estrogens play an important role in skeletal homeostasis, and hormonal deficiency in the ovaries is one of the greatest risk factors of osteoporosis [17]. The ingestion of estrogen has been reported to have an effect on the prevention of bone fractures by increasing the level of inorganic matter in bone and the composition of collagenous fibers [38]. Estrogen is also known to affect bone resorption [18]. In the OVX group, significant increases in blood osteocalcin and blood ALP levels, which are active indicators of osteoblasts, were observed compared with those in the sham group; furthermore, significant decreases in osteocalcin and ALP levels were observed in the RHEC dose group. RE increases BMD, bone minerals, and cancellous bone mass, indicating that red clover affects bone metabolism [17, 25, 39]. It also appears to reduce bone loss by reducing bone turnover by inhibiting ALP and bone resorption [40]. HE has also been reported to prevent the reduction of trabecular thickness due to estradiol deficiency and to reduce the number of osteoclasts in the tibial metaphysic [41]. CTX-1 is a bone resorption marker produced by the activity of osteoclasts [42]. Our findings show that the 3 : 1 complex inhibits bone resorption. It was confirmed that blood ALP, osteocalcin, and CTX-1 were reduced in the RHEC group, suggesting that RHEC could improve bone-related symptoms.

Decreased estrogen levels in blood increase antioxidant stress [43], and these changes are associated with increased climacteric symptoms and increased CVD risk [44]. In this experiment, it was confirmed that oxidative stress was reduced by the administration of RHEC. Antioxidant activity due to red clover and HE has been reported as one of the mechanisms by which RHEC administration alleviates menopausal symptoms [45, 46].

An increase in endothelin-1 and a decrease in NO are representative symptoms of endothelial dysfunction, which are also observed in estrogen-deficient women and ovariectomized animals. Estrogen therapy can protect against this imbalance in endothelial function and diminish the risk of CVD [47, 48]. In the present study, endothelial function was normalized by RHEC administration similar to the case of estrogen administration; it is thus expected to have an effect of reducing the risk of CVD following menopause. Moreover, we identified a synergistic effect of the components of RHEC using HUVECs.

Hormone therapy (HT) used to be the standard of care for managing vasomotor symptoms (VMS) and for preventing chronic diseases until the publication of the Women's Health Initiative in 2002. At present, HT is only approved for four conditions [43]: VMS management, menopausal bone loss, and vulvovaginal and genitourinary symptoms in women <60 years old who are within 10 years of menopause and have no contraindications. Otherwise, the use of HT for the primary prevention of other chronic conditions such as CHD, breast cancer, and dementia remains controversial [44]. The estrogen hormone and estrogenic photochemical usually act via estrogen receptors (ER). These have shown agonistic action with high binding affinity for ER*α* (highly expressed in cancer-sensitive tissues) [49] and ER*β*; isoﬂavones retain selective affinity for ER*β*, which is present in tissues requiring certain stimulation by estrogen to function normally (e.g., bone tissue, bone marrow, adipose, brain, kidney, endothelial cells, and liver) [50]. The binding affinity to ER of RHEC was tested and it was seen that RHEC had more selective affinity to ER*β* (affinity ratio ER*α* : ER*β* = 1 : 1.2). Therefore, when studying phytoestrogen or other estrogenic phytochemicals, in addition to the positive effects of estrogen, there is also a need to assess the risks of proliferation and bleeding of uterine tissue and to demonstrate safety. In this study, it was determined that RHEC had no histological effects on uterine tissues when OVX endometrial tissues were administered it for 12 weeks. Therefore, RHEC can act as an alternative to the currently used HT in improving women's menopausal symptoms.

## 5. Conclusion

In conclusion, RHEC appears to have high potential for comprehensively improving various symptoms of menopause. Further studies on its human application and mechanism of action are needed. Furthermore, more investigation is needed on how RE and HE interact with the inhibition of fat accumulation and the relief of parasympathetic nervous system symptoms improve the lipid profile and correct bone metabolism. Nonetheless, it is expected to be useful as a new alternative for treating menopause.

## Figures and Tables

**Table 1:** Biometric changes after 12 weeks of RHEC administration to OVX rats.

Group	Sham	OVX	RHEC (125)	RHEC (250)	RHEC (500)
Body weight gain (g)	80.74 ± 28.43^c^	169.50 ± 32.26	138.30 ± 12.9^a^	130.31 ± 20.76^a^	126.53 ± 17.56^a^
Visceral fat (g)	3.42 ± 1.82^c^	7.84 ± 1.40	5.73 ± 1.53	5.21 ± 1.41^a^	4.74 ± 0.74^b^
Retroperitoneal fat (g)	3.23 ± 1.39^c^	8.16 ± 2.17	6.29 ± 1.88	5.78 ± 0.82^a^	5.27 ± 0.58^c^
Liver (g)	6.75 ± 0.87	7.69 ± 0.66	6.75 ± 0.37	6.96 ± 0.73	6.67 ± 0.67
Kidney (g)	1.64 ± 0.14	1.68 ± 0.09	1.64 ± 0.19	1.60 ± 0.07	1.58 ± 0.19
Uterus (g)	0.54 ± 0.06^a^	0.11 ± 0.02	0.09 ± 0.01	0.11 ± 0.02	0.11 ± 0.01
Uteric epithelial height (µm)	27.71 ± 5.36^a^	14.34 ± 2.78	15.12 ± 1.97	14.30 ± 2.63	14.97 ± 2.59
Feed intake (g/day)	16.47 ± 1.07^c^	19.98 ± 1.17	18.23 ± 0.89^a^	18.61 ± 0.75	18.38 ± 1.25

Values are expressed as mean ± SD (*n* = 7). OVX, ovariectomy; RHEC, combination of red clover extract and hops extract; FER, food efficiency ratio. ^a^*P* < 0.05 versus OVX; ^b^*P* < 0.01 versus OVX; ^c^*P* < 0.001 versus OVX.

**Table 2:** Results of serum analysis after 12 weeks of RHEC administration to OVX rats.

Group	Sham	OVX	OVX		
			RHEC (125)	RHEC (250)	RHEC (500)
ALT (U/L)	35.8 ± 10.6	37.0 ± 2.5	38.8 ± 5.9	36.9 ± 4.1	35.3 ± 2.9
AST (U/L)	72.5 ± 17.1	78.0 ± 5.6	85.9 ± 8.3	79.0 ± 8.3	72.9 ± 4.9
T-Chol (mg/dL)	76.0 ± 14.7^c^	123.7 ± 17.6	117.1 ± 14.1	109.6 ± 9.0	93.6 ± 10.4^c^
TG (mg/dL)	69.1 ± 16.4	100.4 ± 38.7	78.0 ± 23.8	73.1 ± 10.2	65.7 ± 14.5
HDL-C (mg/dL)	58.5 ± 10.6^a^	73.0 ± 6.1	81.2 ± 8.8	79.4 ± 6.3	67.7 ± 7.4
LDL-C (mg/dL)	6.8 ± 109^c^	13.0 ± 1.8	15.2 ± 2.4	13.6 ± 1.2	11.4 ± 1.1
ALP (U/L)	41.1 ± 10.6^c^	69.7 ± 9.8	74.0 ± 17.6	58.1 ± 11.2	52.9 ± 5.1
Osteocalcin (pg/ml)	771.4 ± 322.9^b^	1723.3 ± 153.6	1743.3 ± 425.5	1498.1 ± 267.8	1265.1 ± 127.9
CTX-1 (pg/ml)	7.15 ± 1.64^c^	15.40 ± 3.90	11.81 ± 2.63^a^	11.53 ± 1.30^b^	6.48 ± 2.02^c^
SOD (U/ml)	42.625 ± 12.856^a^	17.423 ± 14.266	37.263 ± 22.755	46.711 ± 11.368^a^	48.704 ± 14.952^b^
MDA (*µ*M/ml)	10.24 ± 3.45^b^	19.22 ± 3.86	14.65 ± 5.19	13.74 ± 3.50	11.54 ± 4.61^a^

Values are expressed as mean ± SD (*n* = 7). OVX, ovariectomy; RHEC, combination of red clover extract and hops extract. ^a^*P* < 0.05 versus OVX; ^b^*P* < 0.01 versus OVX; ^c^*P* < 0.001 versus OVX.

## Data Availability

Our data are from the analysis of articles and can be provided when necessary.
